# Correlates of emotional intelligence among Lebanese adults: the role of depression, anxiety, suicidal ideation, alcohol use disorder, alexithymia and work fatigue

**DOI:** 10.1186/s40359-021-00525-6

**Published:** 2021-01-28

**Authors:** Sahar Obeid, Chadia Haddad, Kassandra Fares, Diana Malaeb, Hala Sacre, Marwan Akel, Pascale Salameh, Souheil Hallit

**Affiliations:** 1 Research and Psychology Departments, Psychiatric Hospital of the Cross, P.O. Box 60096, Jal Eddib, Lebanon; 2 Faculty of Arts and Sciences, Holy Spirit University of Kaslik (USEK), Jounieh, Lebanon; 3 INSPECT-LB: Institut National de Santé Publique, Epidémiologie Clinique et Toxicologie - Liban, Beirut, Lebanon; 4 Université de Limoges, UMR 1094, Neuroépidémiologie Tropicale, Institut d’Epidémiologie et de Neurologie Tropicale, GEIST, 87000 Limoges, France; 5 School of Pharmacy, Lebanese International University, Beirut, Lebanon; 6 Life Sciences and Health Department, Paris-Est University, Paris, France; 7 Faculty of Medicine, University of Nicosia, Nicosia, Cyprus; 8 Faculty of Pharmacy, Lebanese University, Beirut, Lebanon; 9 Faculty of Medicine and Medical Sciences, Holy Spirit University of Kaslik (USEK), Jounieh, Lebanon

**Keywords:** Emotional intelligence, Depression, Anxiety, Suicidal ideation, Alcohol use disorder, Alexithymia, Lebanon

## Abstract

**Background:**

Relationship between emotional intelligence and emotional variables such as stress, depression, anxiety and mental health has been well documented in child and adult samples. New insights into the association between emotional intelligence and different components of mental health in one study (cognitive, emotional and behavioral dimensions) can help patients, therapists, relatives, and friends to understand, explain, and cope with symptoms. There have been no studies assessing the association between the emotional intelligence (EI) with various factors in Lebanon. This study principal aim was to evaluate how EI is related to mental health issues: social anxiety, depression, alcohol use disorders (AUD), work fatigue, stress and alexithymia in Lebanon.

**Methods:**

789 participants were enrolled in a cross-sectional study between November 2017 and March 2018. A cluster analysis was used to evaluate participants’ profiles with the help of emotional intelligence subscales, to separate the Lebanese population into equal limited units with different characteristics using the K-mean technique.

**Results:**

Three clusters were computed dividing participants into low EI (cluster 1; 24.5%), moderate EI (cluster 2; 43.7%) and high EI (cluster 3; 31.7%) respectively. Fitting into the cluster 1 (low EI) was significantly associated with higher AUD, alexithymia, anxiety, depression, perceived stress, social phobia, emotional, mental and physical work fatigue, suicidal ideation compared to cluster 3 (high EI). Fitting into the cluster 2 (moderate EI) was significantly correlated with higher AUD, depression, alexithymia, anxiety, perceived stress, social phobia, mental work fatigue and suicidal ideation compared to cluster 3 (high EI).

**Conclusion:**

This study results suggest that emotional intelligence is related to different variables, warranting interventions to limit/decrease alcohol abuse and mental/psychological illnesses as much possible.

## Background

Emotional Intelligence (EI) has been described many times in psychological theories. Olivier Serrat (2017) describes EI as “the ability, capacity, skill, or self-perceived ability to identify, assess, and manage the emotions of one’s self, of others, and of groups”. Lyusin [1] also mentioned that feelings and the derivative knowledge of emotions help mankind react better with others. This idea permits individuals to define emotions, understand the importance that imposes one sentiment to another, and convert that information as a source of decision-making [1]. Petrides and Furnham believe that EI is multifactional, meaning that EI is more of a social or character variable rather than a cognitive one that is all about information handling and collecting [2, 3]. Emotional intelligence is commonly defined by four attributes [4]: (1) Self-management—ability to control impulsive feelings and behaviors, to manage emotions in healthy ways; (2) Self-awareness—ability to recognize our own emotions and how they affect thoughts and behavior; (3) Social awareness—capacity to have empathy; (4) Relationship management/social skills– skills to develop and maintain good relationships, communicate clearly, inspire and influence others, and manage conflict. EI is thus an important parameter that affects all aspects of a person's life and conceptualizes the perception, processing, regulation, and management of emotions in self and others [5].

The concept of EI has grown quickly in recent years and has a strong relationship with mental health [6]. Thereby, the intelligent use of emotions is considered essential for one’s physical health and psychological adaptation [7]. Woolery and Salovey (2004) have projected EI as a potential risk factor or protective factor in mental health [7]. Among reported negative consequences, it appears that deficiency in emotional intelligence can have a variety of detrimental psychosocial and physical outcomes for individuals that may require therapeutic intervention*.* Various research have studied the relation between EI and physical/mental health [8, 9], between emotional intelligence and emotional variables such as depression and anxiety [10, 11]; higher emotional intelligence leads to positive quality of life while people with low emotional intelligence tend to develop psychopathology and mental health problems [12]. However, there are no studies that have taken multiple components of mental health in one research.

At the level of depression, Downey et al. witnessed connections between emotional management and control and depression severity [13]. This outcome supported the concept linking both the deficiency of emotional control and the failure to adjust feelings to clinical depression [14, 15]. In addition, Generalized Social Phobia (GSP) is described as an evident fear of numerous social engagements. Individuals with GSP exhibit atypical neural responses to emotional stimuli and regularly show analysis bias in such circumstances. This standpoint may have proposed that GSP implicates difficulties in correctly remarking, understanding, using and handling sentiments [16].

As for alcohol use disorders (AUD), the lack of EI has been identified as a potential correlate of alcohol abuse in adolescents and adults [17]. Actually, studies have shown that inter- and intrapersonal emotional decoding deficits are present in individuals with AUD [18]. In 2007, Goldstein et al. [19] published an article correlating AUD with antisocial syndrome, leading to major relationship problems. It is for this reason that treatment of the regulation of emotions may be in favor of reducing relapses [20, 21].

Previous research [22, 23] has also shown the existence of an adverse and noteworthy relationship between EI and exhaustion. In other words, subjects who have a high level of emotional intelligence are socially active, suffer less body pain, and are therefore less likely to develop burnout syndrome. As for stress, previous research [24, 25] indicated that subjects with high emotional intelligence have a great ability to regulate and express their own emotions, as well as an ability to decode the emotional states of others. Thus, the probability of subjects being overpowered by stress and developing mental health problems is low [26]. Finally, studies stated a negative association between EI and alexithymia [15, 27] and positively associated with self-esteem [28, 29].

Given the theoretical importance of EI in predicting psychological adjustment, it is expected to find a strong correlation between emotional intelligence and general psychological well-being [30, 31]. Relationship between emotional intelligence and emotional variables such as stress, depression, anxiety and mental health has been well documented in child and adult samples [11, 32]. In fact, people with higher emotional intelligence demonstrated less subjective stress, experienced better well-being, and demonstrated better management ability [33]. In addition, it has been demonstrated the value of EI in mood health, stress, psychosomatic health [34] and psychological well-being [35]; thus, the importance of EI depends on its ability to predict good and bad events of life. Therefore, new insights into the association between emotional intelligence and different components of mental health in one study (cognitive, emotional and behavioral dimensions) can help patients, therapists, relatives, and friends to understand, explain, and cope with symptoms. So far, research has pointed out that regulating emotions is the most important dimension of emotional competence affecting mental health. Therefore, the ability to understand emotional information is another crucial factor in daily functioning health and may be a therapeutic goal aimed at developing new programs that incorporate interventions to improve emotional skills [36].

Culturally, Ang et al. proposed that a person might be emotionally intelligent in one culture, but not in another [37]. The values and standards of each person decide the central importance in one self’s lifetime and affect the approach in which emotions are used, identified and assessed [37]. Meta-analytical proof also proposes that traditional values and beliefs have an impact on feelings, insights and intellectual schemas [38]. This suggests that the cultural background influences the EI and can therefore be considered as a precursor of EI. For example, Yurie Igarashi [39] compared two cultural factors: individualism in America and collectivism in India. Results suggested that an individual tendency to individualism is linked to happiness experience in the first country. While an individual tendency to collectivism is linked to happiness experience in the second country [39]. Furthermore, collectivism seems to be positively linked with the use of emotions, the regulation of emotions and the emotional appraisal of others [40]. Lebanon presents a collectivistic culture where citizens are expected to interconnect with the society peers. Consequently, individual emotions are recognized and yet controlled for the profit of the unit*.*

On October 17, 2019, mass protests swept across Lebanon shortly after the government announced new tax measures. In a unique scene, tens of thousands of peaceful protesters from different religious and class sectors of society assembled in cities across the country accusing the political leadership of corruption and calling for social and economic reforms. Underlying frustration with the government and the political elite had been accumulating for years. Public anger has escalated in recent years over electricity and water shortages, as well as the government’s failure to manage the country's waste and economic crises [41]. With the deterioration of the economic, social, political situation, protests, the high rate of poverty and unemployment, it would be important to emphasize the capacities of the Lebanese to manage their own emotions and those of others, and to understand how this management could influence their mental health. As far as we know, there have been no studies assessing the association between the emotional intelligence with various factors in Lebanon. This study principal aim was to establish the profiles of the participants in terms of EI and evaluate the association between EI and mental health issues: social anxiety, depression, alcohol use disorders (AUD), suicidal ideation, burnout, stress and alexithymia in Lebanon.

## Methods

### Sampling and data collection

This study had a cross-sectional design and was conducted between November 2017 and March 2018. Lebanon has two main levels of administrative division: the Mohafazat (district) and the caza (sub-district), which are divided into numerous villages. From a list provided by the Central Agency of Statistics in Lebanon, we chose two villages per Caza where survey documents were given to residents; the latter were arbitrarily selected in a proportionate manner [42]. All adults living in the house were entitled to participate. Those who agreed to take part in the study were invited to complete the questionnaire via a face-to-face interview. Excluded were those with dementia (as reported by a family member). Data assembly was completed by a personnel not related to the study team. The methodology used in this study is similar to the one used in prior publications from the same project [43–50]. The objectives of the papers from this project were different, so were the analyses and the conclusions of each paper. This is the first paper that has the objective of evaluating the association between EI (after dividing participants into clusters) and mental health issues: social anxiety, depression, AUD, suicidal ideation, burnout, stress and alexithymia in Lebanon. Although these studies and the present study utilized the same data set, we have previously used the emotional intelligence variable as part of factors/clusters that would be associated with the other dependent variables (alcohol use disorder, alexithymia, depression, self-esteem, etc.), which is different from the present study's analysis where the clusters according to EI were taken as independent variables.

### Minimal sample size calculation

We set at 50% the predictable occurrence of emotional intelligence among the overall population considering the lack of national studies on this subject. The version 7.2 of the Epi-info software (population survey) estimated at least sample of 384 contributors to guarantee a 95% confidence level.

### Questionnaire

The questionnaire was in Arabic, Lebanon’s native language. Initially, questions about socio-demographic characteristics were included, followed by a list of scales:

### The Quick Emotional Intelligence Self-Assessment

It contains 4 parts: self-management, self-awareness, social awareness and relationship management/social skills. Each part is constituted of 10 interrogations. Greater scores would signify higher EI [51]. Cronbach’s alpha values for each subscale were as follows: 0.888, 0.823, 0.908 and 0.902 respectively.

### The Alcohol Use Disorders Identification Test (AUDIT)

The self-report screening tool has ten elements and evaluates alcohol usage and its consumption habits [52]. Patients having a score equal to or higher than 8 were considered to reflect AUD (α_Cronbach_ = 0.885).

### Toronto Alexithymia Scale (TAS-20)

The TAS-20 [53] usually measures alexithymia through twenty items. Higher scores indicated higher alexithymia [54, 55] (α_Cronbach_ = 0.778).

### Rosenberg self-esteem scale (RSES)

A 10-element scale processes optimistic and pessimistic approaches around one self to assess self-respect [56]. Having higher points meant higher self-worth (α_Cronbach_ = 0.733).

### Hamilton depression rating scale (HDRS)

The validated Arabic version of the HDRS [57] was used in this study [58]. Having higher results designated higher depression (α_Cronbach_ = 0.890).

### Hamilton anxiety scale (HAM-A)

The HAM-A, recently validated in Lebanon [59], is a frequently used tool to indicate the level of anxiety in health care and research places [60]. It contains 14 elements, which show higher anxiety with a higher reached score (α_Cronbach_ = 0.898).

### Evaluation of the Three-Dimensional Work Fatigue Inventory (3D-WFI)

It is composed of 18 questions, each pack of 6 questions measures physical, mental and emotional work fatigue respectively [61]. Higher scores indicate higher fatigue in all 3 dimensions. In this study, the Cronbach alpha for physical work fatigue was 0.823, for mental work fatigue 0.667 and for emotional work fatigue 0.909.

### Columbia–Suicide Severity Rating Scale (C-SSRS)

An instrument to assess suicidal thoughts and comportment. The presence of such ideas is deliberated if the participant replied by “yes” to any question of the 5 asked categories on the C-SSRS [62]. A score of 0 signifies the nonexistence of suicidal ideas (α_Cronbach_ = 0.762).

### The Perceived Stress Scale (PSS)

This tool, composed of 10 items, assesses stress within the previous month. Higher scores show higher levels of stress (α_Cronbach_ = 0.667).

### Liebowitz Social Anxiety Scale (LSAS)

In 1987, Liebowitz established a small scale [63] with the advantage of detecting social anxiety disorder. It is a valid self-report instrument [64]. Items are grouped into 2 subdivisions: 13 intend to report performance-linked anxiety while the other 11 are concerned with social occupations. Cronbach’s alpha values were 0.954 for the whole scale, 0.945 for the fear subscale and 0.953 for the avoidance subscale.

### Translation process

The previously noted scales underwent forward and backward translation. An expert in mental health first converted the English version into the Arabic language. Another expert translated it back to English. A comparison of both English versions helped remove inconsistencies by consensus.

### Statistical analyses

We handled data analysis using the 23rd SPSS software version. Cronbach’s alpha of each scale/subscale was recorded for reliability. A cluster analysis was executed to reveal characteristics of the contributing members as stated by EI subscales and divide people into groups with distinctive profiles utilizing the K-mean method. Analysis permitted for 10 iterations centering results on 0 and convergence was simply assured through a 3-cluster design (i.e. 3 dissimilar profiles). The main benefit of cluster analysis is that similar participants can be grouped together. This helps identify patterns, reveal associations, and outline structure between participants. The emergence of a clear structure out of this analysis can allow easier decision-making. A multivariate analysis of covariance (MANCOVA) was carried out to compare multiple scales scores (being taken as dependent variables) and the 3 clusters, after adjustment over confounding variables (gender, marital status, socioeconomic status, age, children number and education level). A p-value < 0.05 showed significance.

## Results

A total of 789 (83.05%) questionnaires was filled and handed back to the interviewer out of 950 originally distributed. The average age of the participants was 30.30 ± 12.52 years (45.2% females). Other sociodemographic information are arranged in Table 1. In the absence of cutoff values for each of the emotional intelligence subscales, we took the median as a cutoff point. The results showed that 380 (49.4%) had good emotional awareness, 337 (43.8%) had good emotional management, 388 (50.6%) had good social emotional awareness and 406 (51.5%) had good relationship management.

**Table 1:** Sociodemographic characteristics of the sample population

	Frequency (%)
Gender	
Male	423 (54.8%)
Female	349 (45.2%)
Education level	
Illiterate	12 (1.6%)
Primary	39 (5.3%)
Complementary	52 (7.0%)
Secondary	113 (15.2%)
University	462 (62.3%)
Higher education	64 (8.6%)
Socioeconomic status	
< 1000 $	376 (50.7%)
1000–2000 $	260 (35.1%)
> 2000 $	105 (14.2%)
Marital status	
Single	488 (63.1%)
Married	236 (30.5%)
Widowed	19 (2.5%)
Divorced	30 (3.9%)

	Mean ± SD
Age (in years)	30.30 ± 12.52

### Profiles of participants

A cluster analysis, established according to the 4 EI subscales, resulted in three mutual and exclusive clusters: low EI (24.5%), moderate EI (43.7%) and high EI (31.7%) (Table 2).

**Table 2:** Classification of participants in the study sample by cluster analysis according to the emotional intelligence scales

	Cluster 1 N = 188 (24.5%)	Cluster 2 N = 335 (43.7%)	Cluster 3 N = 243 (31.7%)
Emotional awareness score	10.39	19.31	25.28
Emotional management score	10.89	18.82	28.30
Social emotional awareness score	11.44	21.24	30.22
Relationship management score	11.00	20.52	30.96

Cluster 1 = People with low emotional intelligence; Cluster2 = People with moderate emotional intelligence; Cluster3 = People with high emotional intelligence

### Clusters status and correlation with the psychological scales after adjusting for covariates

Higher alcohol use disorder, depression, anxiety and suicidal ideation were significantly found in participants with low EI (cluster1) to moderate EI (cluster 2) compared to high EI (cluster3). Higher alexithymia, perceived stress and social phobia were significantly found in participants with low (cluster 1) vs moderate (cluster 2) EI, low (cluster 1) vs high (cluster 3) EI and moderate (cluster 2) vs high EI (cluster 3). Higher emotional and mental work fatigue were significantly found in participants belonging to cluster 1 (low EI) compared to cluster 2 (moderate EI) and cluster 3 (high EI). A significantly higher physical work fatigue was found in participants belonging to cluster 1 (low EI) compared to cluster 2 (moderate EI) and cluster 3 (high EI). Self-esteem did not differ significantly between clusters (p > 0.05) (Table 3).

**Table 3:** Clusters status and correlation with the psychological scales after adjusting for covariates (age, education level, gender, SES, marital status, number of kids)

Variable	Cluster 1 Low EI	Cluster 2 Moderate EI	Cluster 3 High EI	*F*	P-value (Cluster 1 vs. cluster 2)	P-value (cluster 1 vs. cluster 3)	P-value (Cluster 2 vs. cluster 3)
	Mean (SE)	Mean (SE)	Mean (SE)				
Alcohol dependence	12.03 (0.69)	12.24 (0.47)	7.32 (0.60)	22.248	0.807	**< 0.001**	**< 0.001**
Alexithymia	52.01 (0.95)	55.16 (0.66)	48.71 (0.83)	18.290	**0.007**	**0.011**	**< 0.001**
Self-esteem	24.46 (0.24)	25.04 (0.17)	24.85 (0.21)	1.871	0.054	0.244	0.482
Depression	15.56 (0.85)	15.56 (0.59)	7.01 (0.74)	45.113	0.999	**< 0.001**	**< 0.001**
Anxiety	16.38 (0.84)	18.02 (0.58)	9.26 (0.73)	44.045	0.112	**< 0.001**	**< 0.001**
Perceived stress scale	18.61 (0.57)	20.18 (0.39)	16.26 (0.49)	18.824	**0.024**	**0.002**	**< 0.001**
Social phobia	40.85 (1.90)	45.82 (1.32)	26.63 (1.66)	40.814	**0.033**	**< 0.001**	**< 0.001**
Emotional work fatigue	21.64 (0.65)	16.19 (0.82)	15.58 (0.94)	20.097	**< 0.001**	**< 0.001**	0.637
Physical work fatigue	19.52 (0.51)	16.03 (0.64)	15.48 (0.73)	10.586	**< 0.001**	**< 0.001**	0.553
Mental work fatigue	22.19 (0.59)	15.29 (0.47)	9.83 (0.68)	92.336	**< 0.001**	**< 0.001**	**< 0.001**
Suicidal ideation	0.70 (0.11)	0.70 (0.08)	0.24 (0.10)	7.098	0.964	**0.003**	**< 0.001**

Independent variable is the cluster group. Covariates are: age, education level, gender, SES, marital status, number of kids. Numbers in bold indicate significant p-values. The ANOVA test was used to compare the means of all variables between the three clusters

### Association between the sociodemographic variables and the psychological scales

Higher means AUDIT, emotional work fatigue and suicidal ideation scores were significantly found in male participants compared to females. However, a significantly higher mental work fatigue, relationship management, social emotional awareness and emotional management appeared in male participants compared to females. A significantly higher AUDIT score was found in participants with a primary and complementary compared to university education level. A significantly higher mental work fatigue, relationship management and social emotional awareness were visible in those with a primary vs university education level. Also, a significantly higher alcohol dependence and depression were found in participants with high income as compared to low income. However, a significantly higher alexithymia was found in participants with low as compared to intermediate income. A significantly higher alexithymia and perceived stress were found in single participants as compared to the married one. In addition, higher depression and anxiety scores were significantly and positively correlated with increased age (Table 4).

**Table 4:** Association between the sociodemographic variables and the psychological scales

	AUDIT score	TAS-20 score	RSES	HDRS	HAM-A	PSS	LSAS	Emotional work fatigue	Physical work fatigue	Mental work fatigue	Suicidal ideation	EAS	EMS	SEAS	RMS
	M ± SD	M ± SD	M ± SD	M ± SD	M ± SD	M ± SD	M ± SD	M ± SD	M ± SD	M ± SD	M ± SD	M ± SD	M ± SD	M ± SD	M ± SD
Gender															
Male	12.13 ± 8.38	52.34 ± 10.84	24.58 ± 3.10	12.84 ± 10.56	14.67 ± 10.17	18.37 ± 5.96	39.74 ± 22.54	18.85 ± 10.94	15.96 ± 6.43	14.96 ± 8.75	0.71 ± 1.39	18.60 ± 7.49	19.27 ± 8.35	20.80 ± 8.54	20.48 ± 8.99
Female	8.19 ± 7.48	52.02 ± 9.89	24.60 ± 2.68	12.36 ± 10.29	13.69 ± 9.96	18.71 ± 5.95	39.69 ± 23.35	16.82 ± 10.47	15.55 ± 6.69	16.97 ± 8.98	0.42 ± 1.02	19.62 ± 7.34	20.76 ± 8.32	23.00 ± 8.29	22.92 ± 8.39
p-value	**< 0.001**	0.675	0.903	0.536	0.178	0.426	0.974	**0.014**	0.42	**0.003**	**0.001**	0.061	**0.015**	**< 0.001**	**< 0.001**
Education level															
Illiterate	15.83 ± 10.57	54.91 ± 12.04	24.50 ± 2.02	17.25 ± 8.10	21.66 ± 9.91	18.58 ± 5.01	46.80 ± 11.64	21.50 ± 15.55	14.46 ± 5.57	12.90 ± 6.04	0.75 ± 1.54	14.33 ± 4.73	14.75 ± 5.95	17.83 ± 6.82	18.08 ± 6.59
Primary	14.48 ± 8.12	54.35 ± 9.34	24.51 ± 2.31	15.13 ± 10.58	15.38 ± 8.59	19.05 ± 6.14	40.69 ± 16.81	17.08 ± 9.10	14.60 ± 6.11	10.31 ± 8.06	0.71 ± 1.27	16.97 ± 9.02	16.71 ± 8.01	17.76 ± 8.59	17.68 ± 8.56
Complementary	13.01 ± 8.00	53.34 ± 10.04	25.09 ± 2.01	14.18 ± 10.66	16.76 ± 10.53	18.76 ± 6.18	44.89 ± 22.35	21.02 ± 11.95	16.19 ± 6.49	14.27 ± 8.76	0.62 ± 1.15	17.72 ± 7.21	17.64 ± 8.86	20.02 ± 9.06	18.56 ± 8.52
Secondary	10.29 ± 8.26	52.14 ± 12.17	24.08 ± 3.67	13.42 ± 9.67	14.41 ± 10.23	18.74 ± 6.03	38.52 ± 22.87	19.17 ± 11.32	16.90 ± 6.69	17.21 ± 8.23	0.58 ± 1.27	19.43 ± 6.59	20.27 ± 8.02	22.01 ± 8.30	22.84 ± 8.41
University	9.16 ± 7.48	51.79 ± 10.19	24.70 ± 2.67	11.41 ± 10.15	13.35 ± 10.09	18.35 ± 6.12	39.00 ± 24.19	17.23 ± 10.93	16.09 ± 6.64	16.15 ± 9.13	0.54 ± 1.26	19.16 ± 7.29	20.54 ± 8.35	22.32 ± 8.46	22.06 ± 8.81
Higher education	10.51 ± 9.85	51.69 ± 10.95	24.32 ± 4.02	11.84 ± 11.30	13.53 ± 10.42	18.48 ± 5.03	39.49 ± 22.23	17.42 ± 7.36	17.91 ± 7.55	17.33 ± 8.87	0.44 ± 0.93	20.22 ± 8.11	19.76 ± 8.69	23.09 ± 8.65	22.22 ± 8.48
p-value	**< 0.001**	0.575	0.287	**0.035**	**0.019**	0.972	0.551	0.139	0.058	**0.001**	0.898	**0.041**	**0.005**	**0.006**	**0.002**
Socioeconomic status															
< 1000 $	9.60 ± 7.82	53.48 ± 10.30	24.54 ± 2.89	11.58 ± 9.37	13.90 ± 9.99	18.82 ± 5.78	39.62 ± 22.70	17.76 ± 11.43	14.57 ± 6.03	15.60 ± 8.93	0.52 ± 1.20	19.54 ± 7.40	20.30 ± 8.35	22.30 ± 8.42	22.39 ± 8.67
1000–2000 $	11.13 ± 8.45	50.77 ± 10.64	24.67 ± 2.54	13.25 ± 11.08	13.96 ± 10.06	18.02 ± 6.17	41.18 ± 23.42	17.72 ± 10.25	15.79 ± 6.25	15.66 ± 8.95	0.58 ± 1.19	18.62 ± 7.18	19.39 ± 7.97	21.30 ± 8.44	20.64 ± 8.84
> 2000 $	11.83 ± 8.71	51.53 ± 10.65	24.68 ± 3.40	15.76 ± 12.19	15.90 ± 10.66	17.58 ± 5.66	40.30 ± 22.14	19.26 ± 10.44	16.15 ± 6.93	17.49 ± 8.56	0.83 ± 1.53	18.84 ± 8.13	20.57 ± 9.18	21.16 ± 8.32	21.39 ± 9.12
p-value	**0.012**	**0.005**	0.812	**0.001**	0.182	0.085	0.728	0.432	0.108	0.15	0.096	0.29	0.316	0.252	0.051
Marital status															
Single	10.41 ± 8.23	52.83 ± 10.46	24.59 ± 2.93	12.51 ± 10.58	14.17 ± 10.13	18.88 ± 5.97	39.91 ± 23.70	17.98 ± 1.11	15.67 ± 6.66	15.55 ± 8.85	0.60 ± 1.26	18.98 ± 7.42	19.90 ± 8.33	21.84 ± 8.32	21.60 ± 8.73
Married	10.27 ± 8.17	50.45 ± 10.19	24.51 ± 2.93	13.05 ± 10.34	14.22 ± 10.05	17.60 ± 5.70	39.24 ± 20.94	17.71 ± 9.87	15.89 ± 6.32	16.29 ± 9.05	0.55 ± 1.21	19.01 ± 7.60	19.82 ± 8.44	21.28 ± 8.97	21.24 ± 9.01
p-value	0.83	**0.003**	0.725	0.517	0.949	**0.006**	0.726	0.759	0.689	0.309	0.582	0.951	0.898	0.401	0.609
	r	r	r	r	r	r	r	r	r	r	r	r	r	r	r
Age (in years)	0.067	− 0.051	− 0.038	0.113	0.095	− 0.065	0.019	0.011	− 0.003	0.028	0.001	− 0.043	0.007	− 0.022	− 0.021
p-value	0.064	0.161	0.288	**0.002**	**0.008**	0.075	0.629	0.772	0.94	0.461	0.97	0.241	0.859	0.545	0.568

*AUDIT* The Alcohol Use Disorders Identification Test, *TAS* Toronto Alexithymia Scale, *RSES* Rosenberg self-esteem scale, *HDRS* Hamilton depression rating scale, *HAM-A* Hamilton anxiety scale, *PSS* Perceived Stress Scale, *LSAS* Liebowitz Social Anxiety Scale, *EAS* emotional awareness scale, *EMS* emotional management scale, *SEAS* social emotional awareness scale, *RMS* relationship management scale, *M* mean, *SD* standard deviation; the Student t-test was used to compare the means of two groups (e.g. gender, marital status); the ANOVA test was used to compare the means of three or more groups (e.g. education level and socioeconomic status). r: Pearson correlation coefficient; the Pearson correlation test was used to correlate two continuous variables (e.g. age)

Numbers in bold indicate significant p-values

Post hoc analysis for education level: AUDIT score: university vs primary p = 0.001; university vs complementary p = 0.014

HDRS: no significant associations for education level

HAMA: no significant associations for education level

Burnout depersonalization: secondary vs primary p = 0.001; university vs primary p = 0.003; higher education vs primary p = 0.003

EAS: no significant associations for education level

EMS: no significant associations for education level

SEAS: university vs primary p = 0.023; higher education vs primary p = 0.034

RMS: secondary vs primary p = 0.025; university vs primary p = 0.042

Post hoc analysis for socioeconomic status: AUDIT score: low (< 1000$) vs high (> 2000$) p = 0.042

TAS score: low vs intermediate (1000—2000$) p = 0.004

HDRS: low vs high p = 0.001

### Multivariate analysis

The MANCOVA analysis was performed taking the scales as the dependent variables and the three clusters as an independent variable, adjusting for the covariates (sociodemographic variables).

Fitting into the first cluster (low EI) was highly related to greater AUD (Beta = 4.71), alexithymia (Beta = 3.29), depression (Beta = 8.55), anxiety (Beta = 7.11), perceived stress (Beta = 2.35), social phobia (Beta = 14.22), emotional (Beta = 0.60), physical (Beta = -3.55) and mental work fatigue (Beta = 12.36), and suicidal ideation (Beta = 0.46) compared to cluster 3 (high EI).

Fitting into the second cluster (moderate EI) was highly related to greater AUD (AUDIT score) (Beta = 4.92), alexithymia (Beta = 6.44), depression (Beta = 8.55), anxiety (Beta = 8.75), perceived stress (Beta = 3.92), social phobia (Beta = 19.19), mental work fatigue (Beta = 6.90) and suicidal ideation (Beta = 0.46) compared to cluster 3 (Table 5).

**Table 5:** Multivariate analysis of covariance (MANCOVA)

	Beta	*p*	95% confidence Interval	
			Lower	Upper
Alcohol use disorder (AUDIT score)				
Gender (females versus males*)	− 2.623	**< 0.001**	− 3.963	− 1.282
Number of kids	− 1.052	**0.012**	− 1.875	− 0.229
University vs illiterate* level of education	− 4.611	**0.002**	− 7.473	− 1.749
Cluster 1 (People with low emotional intelligence) vs cluster 3 (high emotional intelligence)*	4.718	**< 0.001**	2.887	6.548
Cluster 2 (People with moderate emotional intelligence) vs cluster 3 (high emotional intelligence)*	4.924	**< 0.001**	3.401	6.447
Alexithymia (TAS score)				
Cluster 1 (People with low emotional intelligence) vs cluster 3 (high emotional intelligence)*	3.299	**0.011**	0.764	5.834
Cluster 2 (People with moderate emotional intelligence) vs cluster 3 (high emotional intelligence)*	6.448	**< 0.001**	4.339	8.557
Self-esteem				
Cluster 1 (People with low emotional intelligence) vs cluster 3 (high emotional intelligence)*	− 0.388	0.244	− 1.043	0.266
Cluster 2 (People with moderate emotional intelligence) vs cluster 3 (high emotional intelligence)*	0.195	0.482	− 0.350	0.739
Depression				
High vs low* SES	4.909	**0.039**	0.243	9.575
Cluster 1 (People with low emotional intelligence) vs cluster 3 (high emotional intelligence)*	8.551	**< 0.001**	6.291	10.811
Cluster 2 (People with moderate emotional intelligence) vs cluster 3 (high emotional intelligence)*	8.550	**< 0.001**	6.670	10.430
Anxiety				
Cluster 1 (People with low emotional intelligence) vs cluster 3 (high emotional intelligence)*	7.113	**< 0.001**	4.876	9.350
Cluster 2 (People with moderate emotional intelligence) vs cluster 3 (high emotional intelligence)*	8.754	**< 0.001**	6.892	10.615
Perceived Stress Scale				
Cluster 1 (People with low emotional intelligence) vs cluster 3 (high emotional intelligence)*	2.351	**0.002**	0.839	3.863
Cluster 2 (People with moderate emotional intelligence) vs cluster 3 (high emotional intelligence)*	3.926	**< 0.001**	2.668	5.184
Social phobia				
Cluster 1 (People with low emotional intelligence) vs cluster 3 (high emotional intelligence)*	14.223	**< 0.001**	9.178	19.269
Cluster 2 (People with moderate emotional intelligence) vs cluster 3 (high emotional intelligence)*	19.190	**< 0.001**	14.993	23.387
Emotional work fatigue				
Cluster 1 (People with moderate emotional intelligence) vs cluster 3 (high emotional intelligence)*	5.456	**< 0.001**	3.368	7.544
Cluster 2 (People with low emotional intelligence) vs cluster 3 (high emotional intelligence)*	− 0.603	0.637	− 3.113	1.907
Physical work fatigue				
High vs low* SES	2.89	**0.023**	0.40	5.38
Cluster 1 (People with low emotional intelligence) vs cluster 3 (high emotional intelligence)*	3.550	**< 0.001**	1.597	5.502
Cluster 2 (People with moderate emotional intelligence) vs cluster 3 (high emotional intelligence)*	0.490	0.553	− 1.134	2.114
Mental work fatigue				
High vs low* SES	4.00	**0.001**	1.66	6.33
Cluster 1 (People with low emotional intelligence) vs cluster 3 (high emotional intelligence)*	12.360	**< 0.001**	10.547	14.173
Cluster 2 (People with moderate emotional intelligence) vs cluster 3 (high emotional intelligence)*	6.900	**< 0.001**	5.392	8.408
Suicidal ideation				
Cluster 1 (People with low emotional intelligence) vs cluster 3 (high emotional intelligence)*	0.467	**0.003**	0.159	0.775
Cluster 2 (People with moderate emotional intelligence) vs cluster 3 (high emotional intelligence)*	0.460	**< 0.001**	0.204	0.716

Cluster 1 = People with low emotional intelligence; Cluster2 = People with moderate emotional intelligence; Cluster3 = People with high emotional intelligence

In the global model, the independent variable was the cluster groups and covariates were marital status, age, SES, number of kids, education level and gender

^*^Reference group; Numbers in bold indicate significant p-values

## Discussion

The present study evaluated the association of emotional intelligence with several mental health problems, including depression, alexithymia, social phobia, work fatigue, alcohol consumption problems and stress in Lebanon. Overall, people both in the low and moderate EI clusters had higher odds of AUD, alexithymia, anxiety, depression, tension, social anxiety, exhaustion and suicidal tendency compared to those with high EI.

The results of this study showed that 24.5% of the participants were classified as having low EI, 43.7% moderate EI and 31.7% high EI. These percentages may be discussed based on the culture differences. Several studies have revealed interesting cross-cultural differences in emotionality. For example, North Americans tend to maximize experiencing positive emotions and minimize experiencing negative emotions. However, this tendency is weaker in Asian societies [65]. Oyserman, Coon, and Kemmelmeier [66] proposed that subjects of collectivistic cultures are entertained to regulate their emotional expressions so as to maintain in-group harmony. On the other hand, Markus and Kitayama [67] [67] stated that individualists are stimulated to express their feelings more directly because they do not expect others to read their minds in social exchanges. However, Scott, Ciarrochi, and Deane [68] found that individualism was associated with less skill in managing both self and others emotions. Another study revealed that collectivists inclined to have better perception of emotions in self and others, which in turn, might make them better skilled in managing emotions in both self and others [69]. Therefore, findings of our study suggest that emotion differentiation may increase an individual’s interpersonal adaptability in collectivistic cultures; behaving appropriately in interpersonal situations often requires understanding other’s feelings—a stamp of an emotionally intelligent person [70]. Thus, it may be that adaptive emotional functioning is differently related to individualistic-collectivistic orientations.

The correlation between EI and depression is well documented in the literature [13, 71, 72]. A study had found that EI is a potential risk factor for mental and physical health, especially depression [7]. People with poor EI have a lower emotion clarity and an inability to regulate their own emotional states, in contrast to people with high EI that suffer less stress and demonstrate better management performance [73]. People with low to moderate EI had an inability to control their negative emotions and are more prone to stress and depression [74].

An overlap exists between emotional intelligence and alexithymia and this correlation had been reported in literature [75]. Studies had shown that individuals with high degrees of alexithymia experience low EI [76, 77]. In other words, these individuals face difficulties in identifying emotions in the facial expressions of others and have a limited ability to think about and use emotions to cope with stressful situations. High EI is a protecting factor against social damages, as people can correctly understand, express and use their emotions in their thinking and practice, while people with low EI are confused about their feelings, do not have any ideas about their emotions and have a difficulty expressing themselves [78].

Relationship between emotional intelligence and suicidal ideation had been reported in several studies [79–81]. Numerous studies had identified various factors that may increase or decrease suicidal tendencies such as among those risk factors of suicidal propensities: depression, feeling hopelessness, problems with anger and self-esteem, difficulties in problem solving and expressing emotions [82–84]. People with high emotional intelligence have the ability to cope with stressful life events and are psychologically and physically healthier than individuals with low EI [74]. Negative life experiences of people and the incapacity to tolerate those instances, increase the vulnerability for negative thoughts as well as suicidal ideation [79].

The relation between work fatigue and emotional intelligence can be explained by the fact that people with higher EI, suffer less from the aspects of this syndrome, since they are more active, have less insomnia problems and less complains from body pains and disorders [22, 23]. Social awareness clarifies work fatigue syndrome most. Those people are aware and knowledgeable with slight social tips and connections that reflect others’ requests and wishes [22, 23]. This is a powerful skill, which qualifies people for education, professions and management features and aids them to experience good and effective relationships with others. Also, this aptitude improves contact with social care facilities that plays an important role in minimizing difficulties and conflicts [23].

The findings in this study between emotional intelligence and social phobia relation can be explained by the fact that the capacity to manage and identify social situations is tightly connected to the meaning behind EI [85–88]. Thus, it is reasonable that patients suffering from social anxiety may have deficiencies in emotional intelligence levels. It is possible that social phobia is depicted by an exaggerated terror of social situations that reflects a poor aptitude to ‘read’ these situations, as shown by lower EI. This is in agreement with previous results showing that being socially nervous or timid may present a lower ability to explore social situations, monitor their own emotions, as suggested by an impairment in judging their social performance [85–88].

In this study, people in the low EI category were at a higher risk of stress development since emotional intelligence is highly linked with stress management, problem-solving skills, well-being and mental health [81, 89]. The majority of studies reviewed indicate a relationship between emotional intelligence, stress, coping strategies and health [24, 81]. For example, Pau et al. concluded that people with high emotional intelligence suffered less perceived stress and experienced better health and well-being [24]. In addition, Gohm et al. concluded that emotional intelligence may protect people from stress as high emotional intelligence is related to active coping and better adaptation [90]. The use of emotional intelligence concepts may, therefore, provide insights into strategies to help foster self-engagement in practice and enhance positive attitudes, while decreasing perceived stress.

In our study, there was a close relationship between alcohol use disorder and low emotional intelligence. Evolving emotional intelligence abilities are beneficial in personal life and multiple work domains since it has been recognized to be of significant importance in alcohol abuse potential with adolescents and adults. A previous study revealed that lower EI scores were associated with reduced management strategies, that in turn anticipated a person’s ability to face alcohol dependence [17]. Coping strategies have been previously shown to be linked to different variables such as motivation, self-regulation, self-awareness and social skills, the deficiency of which generally considered as major mediators of alcohol dependence [91]. Consequently, one’s inability to precisely convey, realize, distinguish and control emotional expressions can predict alcohol abuse.

Concerning sociodemographic characteristics and their relationship with the scales used, our results showed that female gender was associated with lower AUD compared to males. As previous studies showed, men were more prone to alcoholism than women [92, 93]. This could be related to the cultural or religious norms and values that render women less at ease in reporting embarrassing or prohibited behaviors. University education level was associated with lower alcohol use disorder compared to illiteracy, as reported by other findings [94]. People with low education and those who left school are more at risk for alcohol disorders and dependence [94].

### Clinical implications

Due to the significant role of EI on subjects’ mental health, healthcare professionals should give a special importance to the role of emotions in the life of each person, encourage avoidance programs and arrange services to learn emotional hacks. Training some of the components of EI such as interpersonal skills, interpersonal awareness, problem solving skills, methods to cope with stress can probably be a way to improve EI overall. In a practical way, the emotional skills of individuals must be specifically trained: (1) Emotional awareness training involves identifying emotional states and recognizing how particular situations can trigger mood changes that influence performance. (2) Guidance on emotional understanding includes considering the way emotional states can guess behavior and thoughts. (3) Preparation for emotional mend denotes that subjects are capable of successfully maintaining positive emotional statuses and fixing negative ones. Therefore, specific training on EI will offer individuals means to handle nervous incidents and negative emotional reactions that commonly occur when dealing with fellow workers, family and partners. This will certainly influence their well-being and quality of life. Future research should evaluate the effectiveness of such strategies/trainings.

### Limitations and strengths

Our study limitations are mostly due to the design of the cross-sectional questionnaire. Larger longitudinal schemes should examine causal sequential relationships while bearing in mind other physical, cognitive, behavioral and sociodemographic factors. Although our sample was randomly selected across Lebanese regions, a selection bias might still exist, which further hinders the generalizability of the results. In addition, the Arabic versions of different scales used have not been validated yet (the C-SSRS scale [95] and the AUDIT scale [96] have been validated among Lebanese adolescents but not adults). However, the project sheds light on various significant outcomes and is the first, as far as we know, to address factors related to emotional intelligence among a descriptive sample composed of Lebanese participants. In other words, this study was able to show an association with EI, including the mental health multicomponent in one study, and results of this study are original, in a way that they replicate the results of other Western countries.

## Conclusion

In conclusion, our study found that there is a link between social anxiety, alcohol dependence, stress, self-esteem, depression, alexithymia, burnout syndrome, and emotional intelligence. This study is very useful as it has demonstrated that emotional intelligence is related to different variables, warranting interventions to limit alcohol abuse, eliminate stress, which will have positive effects on mental health. In future perspectives, we recommend that after applying the intervention strategies to a given population, it would be interesting to reevaluate the results in order to emphasize the feasibility and effectiveness of the strategies implemented.

## Data Availability

The authors do not have the right to share any data information as per the ethics committee.

## Abbreviations

EI: Emotional intelligence
GSP: Generalized social phobia
AUD: Alcohol use disorders
AUDIT: Alcohol use disorders identification test
TAS: Toronto Alexithymia Scale
RSES: Rosenberg Self-Esteem Scale
HDRS: Hamilton Depression Rating Scale
HAM-A: Hamilton Anxiety Scale
3D-WFI: Three-dimensional work fatigue inventory
C-CSSRS: Columbia-Suicide Severity Rating Scale
PSS: Perceived Stress Scale
LSAS: Liebowitz Social Anxiety Scale
MANCOVA: Multivariate analysis of covariance
